# Supplementary material for: Automatic cassava disease recognition using object segmentation and progressive learning
Source: PeerJ Comput Sci. 2025 Mar 18;11:e2721. doi: 10.7717/peerj-cs.2721 (PMC11935765; doi:10.7717/peerj-cs.2721)
Supplement: Supplemental Information 1 [file peerj-cs-11-2721-s001.pdf]

On-field Cassava Leaves

Cassava is an important staple crop grown in tropical and subtropical areas of the world. However, viral diseases can significantly impact yields and food security for smallholder farmers that rely on cassava. Timely and accurate disease detection in the field is critical but poses

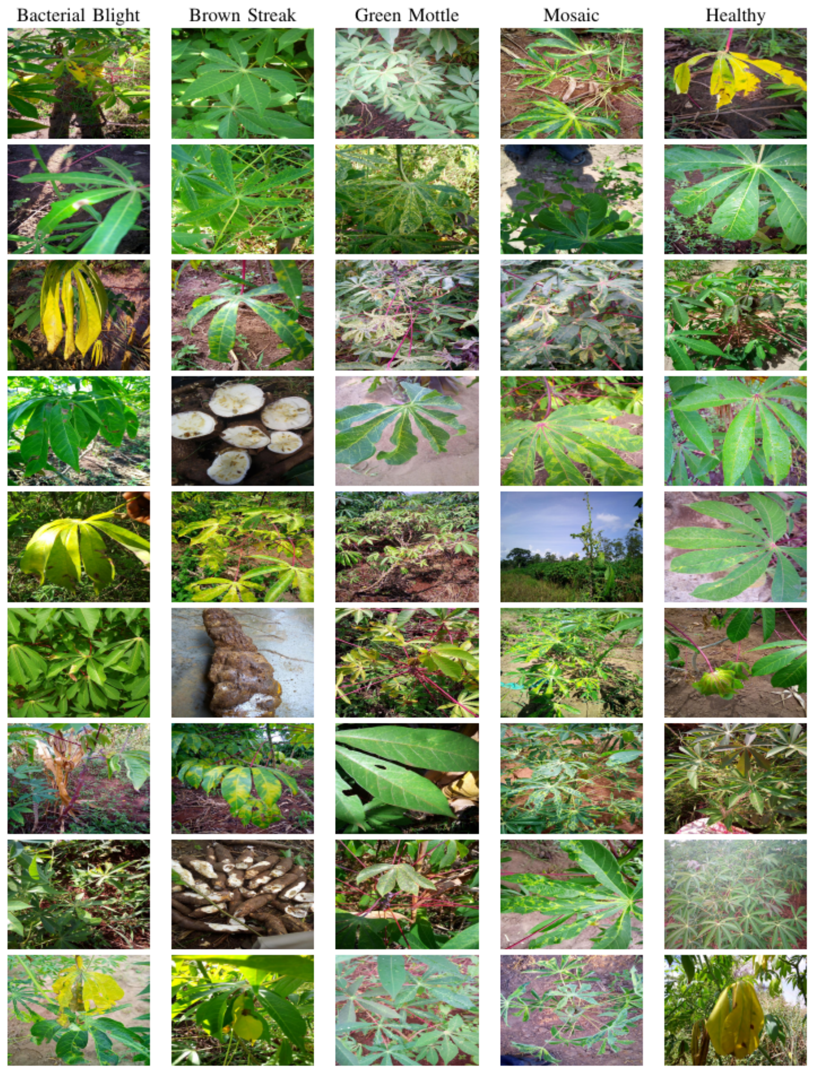

Object Segmentation Model

Detecting diseases from on-field images presents several key challenges: Complex backgrounds with soil, stakes, foliage obstructing leaves; Inconsistent lighting and shadows; Leaf deformation and occlusion

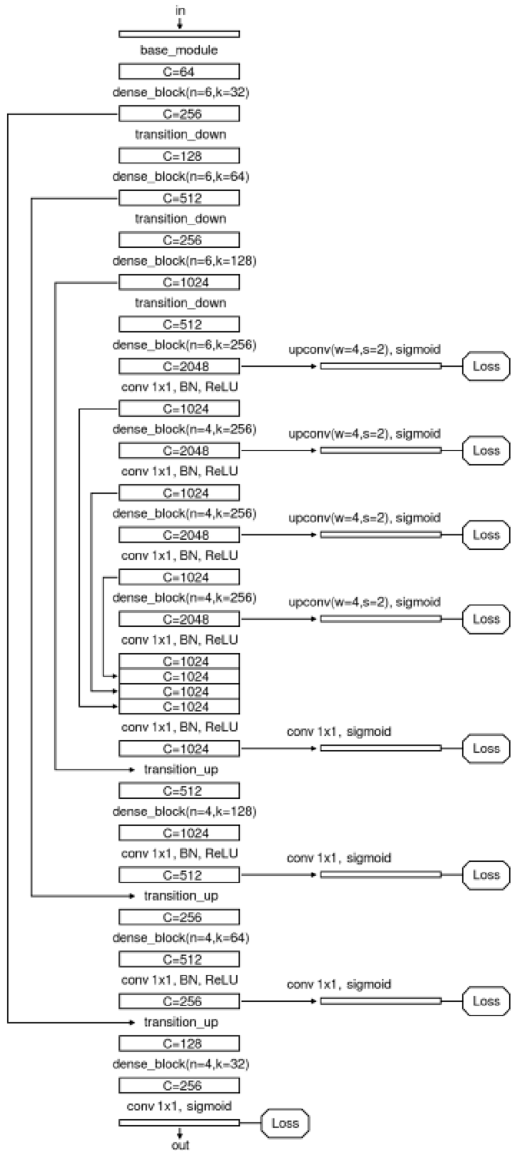

Extracted Cassava Leaves

Before cassava disease recognition step, it is of vital importance to carry out localization of the cassava leaf object from the cluttered background.

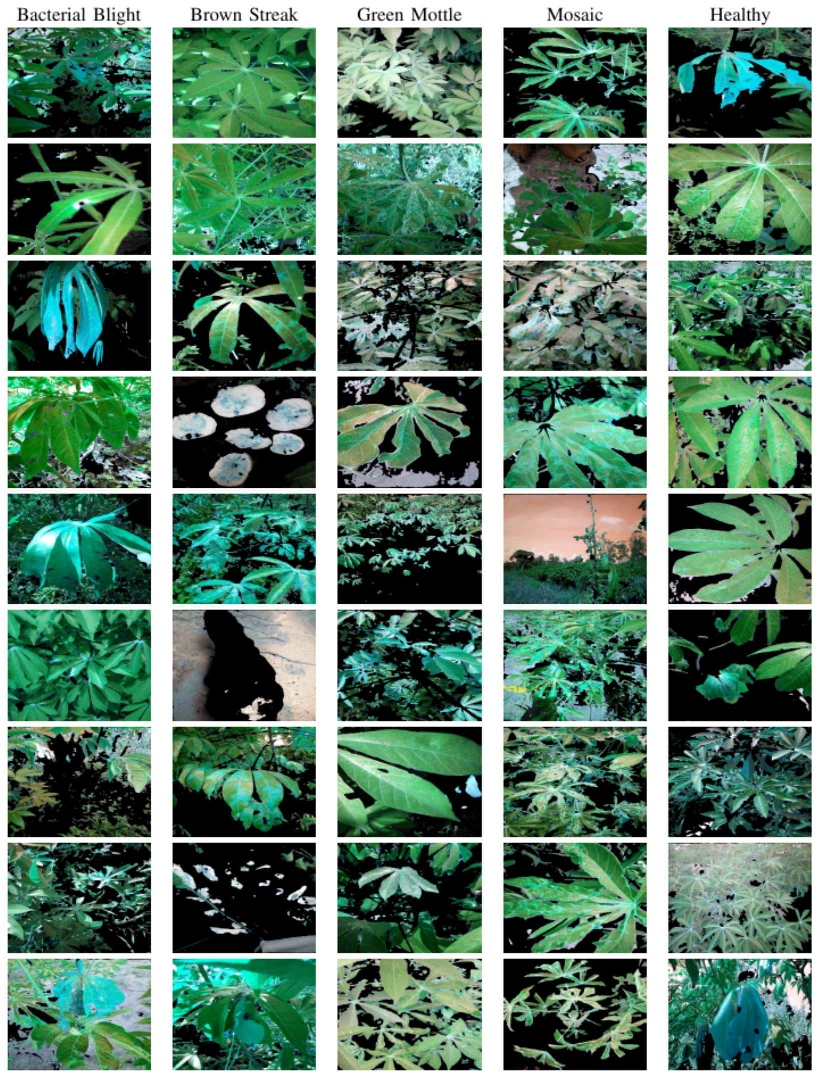

Cassava Disease Recognition

An ensemble network utilizing efficient deep learning networks is proposed to reduce computational costs while enhancing overall performance in image recognition tasks.
The proposed AI-based cassava disease recognition system outperforms traditional microscopic methods significantly in efficiency, with more than 90% accuracy in less than 5 seconds using a Nvidia GTX1080 graphic card for model inference.

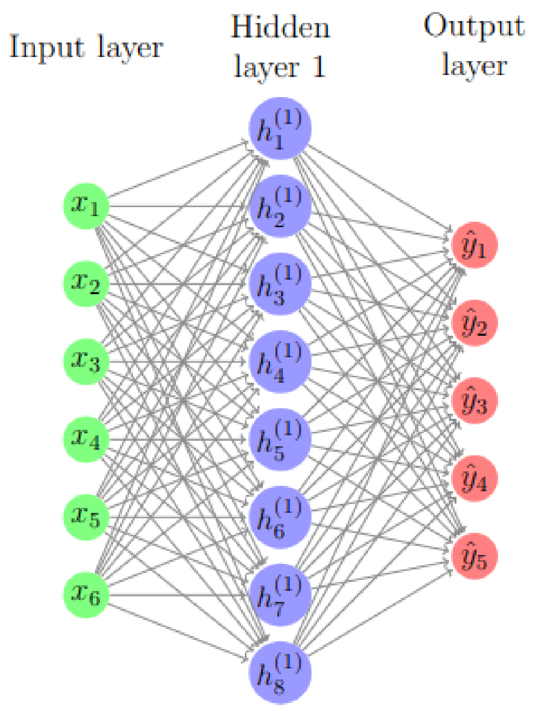

Solution	Public Leaderboard	Private Leaderboard
1st place	91.36%	91.32%
2nd place	90.25%	90.43%
3rd place	90.59%	90.28%
submission (19th)	90.52%	90.11%
late submission	**91.38%**	**91.43%**
